# The Social and Professional Image of the Nurse: Results of an Online Snowball Sampling Survey among the General Population in the Post-Pandemic Period

**DOI:** 10.3390/nursrep13030109

**Published:** 2023-09-12

**Authors:** Ivan Rubbi, Roberto Lupo, Alessia Lezzi, Valeria Cremonini, Maicol Carvello, Martina Caricato, Luana Conte, Marcello Antonazzo, Cosimo Caldararo, Stefano Botti, Vincenzo Andretta, Pio Lattarulo, Elsa Vitale

**Affiliations:** 1 School of Nursing, University of Bologna, AUSL Romagna, 40121 Bologna, Italy; 2 ‘San Giuseppe da Copertino’ Hospital, ASL (Local Health Authority) Lecce, 73100 Lecce, Italy; 3 ANT Italia ONLUS Foundation (National Cancer Association), 73100 Lecce, Italy; 4 Brisighella Community Hospital, Azienda USL della Romagna, 48013 Brisighella, Italy; 5 “Istituto per i Servizi alla Persona per l’Europa” (I.S.P.E.R.S.A.), 73043 Copertino, Italy; 6 Laboratory of Biomedical Physics and Environment, Department of Mathematics and Physics “E. De Giorgi”, University of Salento, 73100 Lecce, Italy; 7 Advanced Data Analysis in Medicine (ADAM), University of Salento, 73100 Lecce, Italy; 8 ASL (Local Health Authority) Lecce, 73100 Lecce, Italy; 9 Dipartimento di Scienze e Tecnologie Biologiche ed Ambientali, University of Salento, 73100 Lecce, Italy; 10 Azienda USL-IRCCS Reggio Emilia, 42122 Reggio Emilia, Italy; 11 UOC Programmazione e Controllo di Gestione AOU San Giovanni di Dio e RUGGI d’Aragona, 84131 Salerno, Italy; 12 ASL (Local Health Authority) Taranto, 74121 Taranto, Italy; 13 ASL (Local Health Authority) Bari, 70026 Modugno, Italy

**Keywords:** COVID-19, general population, nurse, professional image

## Abstract

*Background*. The COVID-19 pandemic has transformed people’s lifestyles by imposing social, economic, and labor changes. Health professionals have been in the spotlight, occasionally even elevated to the status of “heroes”, as they have been at the forefront of the health emergency. Media exposure has undoubtedly played a pivotal role in the evolution and perception of the public’s image of nurses, especially within the Italian context. Currently, there is only one study conducted in Italy during the pandemic period. For this reason, we have opted to delve further into this subject during the post-pandemic period, with the ultimate goal of identifying this perceptual evolution. *Methods*. An online snowball sampling survey was conducted among the Italian population using social networks between August 2022 and January 2023. The survey utilized the Nursing Attitude Questionnaire (NAQ) to collect data. *Results*. The sample consisted of 564 individuals. Among the respondents, 63.8% (n = 360) were familiar with the nursing pathway, and the level of agreement regarding the training of nurses was 81.5% (n = 260). In terms of recognizing the professional role of nurses, variations emerged in certain domains of the NAQ. Specifically, professional values of nurses were more pronounced in northern and central Italy (M = 26.27). Moreover, stereotypes were more prevalent in the north (23.73 ± 3.538) and the center (23.13 ± 4.104) (*p* = 0.001). *Conclusions*. The sample acknowledged a unique competence inherent to nurses that cannot be replaced by other professionals. The study population perceives the nursing profession as pivotal within the IHS (Integrated Health System). However, the attractiveness of the profession remains exceedingly low. This study was not registered on a publicly available registry.

## 1. Introduction

The emergence of the COVID-19 pandemic has significantly elevated the prominence and visibility of the nursing profession within both the public eye and mainstream media. During the period of March–April 2020, media coverage pertaining to nurses saw a threefold increase (coinciding with the World Health Organization’s declaration of COVID-19 as a pandemic in March 2020) in comparison to the previous year [1]. The Italian and global media landscape, spanning television, radio, print, and online platforms, remained extensively engaged from February 2020 onwards, offering continuous narratives about the pandemic and its multifaceted aspects. This sustained engagement has led to the reconfiguration of the nurse’s image in the collective consciousness, fostering a heightened awareness and renewed identity [2].

Healthcare professionals emerged as frontline responders, navigating critical scenarios necessitating heightened expertise across diverse care environments. This exposed them to unprecedented risks of infection and emotional strain, surpassing the challenges faced by professionals in other fields, thereby influencing their present actions and potentially shaping future practices [3]. Concurrently, the prevailing image of nurses, often informed by stereotypes [4], has experienced a noteworthy transformation during this historical juncture. Notably, an emblematic dimension of this elevated nursing profile has been the attribution of collective or individual designations such as “angels” or “heroes.” While these labels reflect an intention to acknowledge the courage, compassion, and dedication intrinsic to the profession [5,6], they also present a dynamic wherein society revises the archetype of the “compassionate caregiver”. However, this revision seldom acknowledges the nurse’s autonomy within their professional scope.

A study conducted within Italy, aimed at discerning societal perceptions of the nursing role, underscores the profession’s evolutionary trajectory in recent years. Despite these advancements, a considerable portion of society remains unaware of the nursing profession’s multifaceted role and capabilities [7]. Globally, nursing remains among the most misunderstood occupations, both in the eyes of the media and the public. The scholarly literature advocates for social channels as mechanisms for healthcare professionals to represent and safeguard their vocations. Paradoxically, the improper application of such platforms can lead to adverse outcomes, potentially tarnishing not only the nurse’s image but also the larger healthcare institution to which they belong. This underscores the profound impact of mismanaged image projection and its corresponding repercussions [8]. Bennett et al. [1] underscored the superficiality of media portrayals, wherein labels such as “angels” and “heroes” inadvertently undermine the intricate expertise integral to the nursing role. Consequently, this shift in perception repositions the profession from one rooted in expert practice to one of tireless service [8]. While an underlying respect for nurses among the Italian population is evident, their competencies are often underappreciated. A survey exploring the perception of nurses’ images, supported by the educational transformations introduced through the Bologna Process, contributed an Italian perspective on this matter [9]. In the 2017 study, 398 individuals were interviewed and categorized into four groups: Very Important Persons (VIPs), upper-school students, nursing students, and the general population group [10]. In this work, the results were correlated with demographic characteristics such as age, gender, education, profession, knowledge of the nursing profession, and related educational paths. Additionally, the study explored the varying levels of satisfaction associated with the participants’ previous hospitalization experiences [11,12].

These variables were subsequently examined in connection with other research endeavors related to the perception of the nurse’s image among students in schools that received orientation information about the nursing degree program in Italy [13]. The changes in educational policies, transitioning nursing education to the university level, have contributed to a reasonable shift in the perceived image of nurses, which in turn seems to impact national healthcare policies [13,14].

In Italy, in the aforementioned studies conducted on different samples [13], the Nursing Attitude Questionnaire (NAQ) was employed. The present study aimed to replicate the use of this tool for descriptive purposes, involving the broader Italian general population during the post-pandemic period. This was accomplished through an online survey using social media channels widely spread across the Italian territory, allowing for a more diverse and comprehensive view of the population (albeit limited to those reachable through the employed dissemination channels). The goal was to analyze the population’s perception of the nurse’s image, specifically assessing how this perception had changed in the post-pandemic period due to the increased visibility of nurses during the COVID-19 emergency. To date, only a limited number of studies in the literature have delved into the exploration of the nursing image during both health crisis phases, as posited by Rezaei-Adaryani et al. [10], and the subsequent post-pandemic period: currently, in Italy, another study is underway that assesses the change in the population’s perception of the nurse’s image within the pandemic context (from September 2020 to February 2021), but not within the post-pandemic context.

## 2. Materials and Methods

### 2.1. Study Design

An online snowball sampling survey was conducted among the Italian population until the designated conclusion of the study using social networks.

### 2.2. Participants in the Study

The study encompassed an adult population, enrolling participants within the age range of 20 to 70 years who provided their informed consent for participation. Subjects below the age of 20 and above the age of 70 were excluded from the study. Additionally, individuals who were not part of the Italian population were also excluded. Given that this study had descriptive objectives, a formal sample size calculation was not conducted.

### 2.3. Data Collection and Instruments

Upon securing informed consent from each participant, data collection took place from August 2022 to January 2023. The evaluation of the perceived nurse’s image was facilitated through the utilization of the Nursing Attitude Questionnaire (NAQ). Originally formulated by Hoskins [11] and subsequently adopted by Toth [12], this tool serves to gauge values and sentiments associated with the nurse’s image as perceived by distinct segments of the population, including ordinary citizens, nursing students, and upper-school students. In 1998, Toth reported a Cronbach’s coefficient of 0.75 [12]. Since the questionnaire was not available in Italian, the same questionnaire was translated from English to Italian by two independent external translators, ensuring the preservation of all original elements. This was followed by a back-translation of the reconciled Italian version into English by a native speaker. This translated and back-translated version has been previously used in two Italian studies [9,13]. The questionnaire comprises a set of 30 Likert items, encompassing a range of responses from 1 (strongly disagree) to 5 (strongly agree), with intermediate options of 2 (mildly disagree), 3 (neutral), and 4 (mildly agree). The completion of the questionnaire typically requires approximately ten minutes. The survey delved into five distinct domains, namely:

1. Professional Role (10 items);
2. Professional Values (7 items);
3. Stereotypes (6 items);
4. Professional Activities (4 items);
5. Nursing Professionals’ Attributes and Responsibilities (3 items).

Notably, scores on the NAQ span from 30 to 150 points, wherein higher scores indicate a more favorable attitude towards nursing, whereas lower scores reflect a less positive perception. Prior to statistical analysis, seven items underwent recoding [12]. The questionnaire also included a section containing socio-demographic data: gender (female or male), age (expressed in years), marital status (single, married, cohabiting, separated/divorced, widowed), educational attainment (no degree, elementary school diploma, lower secondary school diploma, high school diploma, university degree, postgraduate education), region of Italy (north, central, or south), religious belief, employment status (student, homemaker, private sector employee, public sector employee, self-employed, retired, unemployed; if the respondent is a student, they must indicate their degree program and the year of enrollment), profession, whether the respondent works in a healthcare profession, whether the respondent has a family member or acquaintance who is a nurse, whether the respondent has ever been hospitalized, and whether the respondent or their family member required hospital and/or home care assistance from a nurse during the COVID-19 emergency, along with their level of satisfaction, and the extent to which TV series and/or news have influenced their perceived image of nurses [9]. The questionnaire was uploaded to the Google Forms platform and made visible using social media.

### 2.4. Data Analysis

The analysis was performed using Jamovi version 2.3.18 and Microsoft Office Excel software. The characteristics of the sample and NAQ scores were subjected to an array of statistical measures, including frequencies, percentages, central tendency indices such as mean and median, as well as measures of dispersion like standard deviation (SD) and range. Statistical significance was ascertained through various methods: ANOVA (Analysis of Variance) and the *t*-test were employed for interval data variables, while the chi-square test was utilized for nominal variables. To examine correlations between variables, Pearson’s coefficient was applied. A significance level of *p* < 0.05 was adopted to determine statistical significance, accompanied by a confidence interval of 95%. The reliability of each test was assessed using Cronbach’s alpha, which measures internal consistency and reliability.

### 2.5. Ethical Considerations

The bioethics committee of the University of Bologna, under protocol number 13221 dated 8 February 2017, had previously granted approval for earlier studies conducted at the university. These prior studies employed the NAQ to gauge the extent of the population’s perception regarding the nurse’s image. The present study repurposes this research within the context of the post-pandemic period of COVID-19, thereby expanding upon the investigations initiated during the pre-pandemic phase. The questionnaire, administered via self-completion by participants through a provided link, was meticulously structured to ensure the compiler’s anonymity. Information was automatically updated in the database in such a manner that the identity of the respondent could not be traced, aligning with confidentiality requirements as stipulated by Law No. 675/1996. Prior to sampling, all necessary information regarding informed consent was included in the introductory section of the questionnaire. Participants’ adherence to the study was voluntary and demonstrated through a designated acceptance prompt on the questionnaire platform.

## 3. Results

Overall, the sample consists of 564 people: 76.9 percent (n = 434) from the south (southern and insular Italy), 13.6 percent (n = 77) from the north (northern Italy), 7.9 percent (n = 45) from the center (central Italy), and 1.4% (n = 8) did not state where they were from.

### 3.1. Description of the Participants

The age group most significantly represented is individuals aged 20 to 30 years, accounting for 34.9% (n = 197) of the sample. This is followed by the 41 to 50 years age group at 22.7% (n = 128) and the 31 to 40 years age group at 20.7% (n = 117). Within the sample, 39.9% (n = 225) identified as single, while 29.8% (n = 168) were married. In terms of educational qualifications, 51.4% (n = 290) possessed a high school diploma, and 28.9% (n = 163) held a bachelor’s degree. Regarding employment, 34.2% (n = 193) were employed in the public sector and 18.8% (n = 106) in the private sector. Notably, 22.7% (n = 128) of the sample were students, with 61.6% (n = 77) studying nursing and 14.4% (n = 18) enrolled in other health-related programs. A substantial proportion of the sample, 71.3% (n = 402), reported having a family member or acquaintance within the nursing profession. Turning to respondents’ firsthand experience in inpatient care, 38.1% (n = 215) did not provide a response to this query, while 27.7% (n = 156) indicated having undergone an inpatient stay within the past decade. However, the entire population that had hospital experiences, 66.2% (n = 235), reported being satisfied with the treatment received.

Out of those who have had experiences as inpatients, 66.2% (n = 235) express satisfaction with the treatment they received. During the pandemic, a significant portion of the respondents, specifically 70.0% (n = 395), did not perceive an increase in the demand for nursing care. However, within the subset of individuals who availed of nursing services, a mere 43.5% (n = 138) expressed satisfaction with the care received. Turning attention to nursing education, a significant majority of respondents, accounting for 63.8% (n = 360), are familiar with the nursing curriculum. Furthermore, the level of agreement regarding the training of nurses stands at 81.5% (n = 260), signifying the prevailing consensus among respondents on this matter.

### 3.2. Nursing Attitude Questionnaire

The internal consistency of the NAQ demonstrates a commendable level (α = 0.893), mirroring the robustness of the instrument’s measurements. Likewise, the sample size is substantial and exhibits a strong suitability for analysis (KMO = 0.908), as confirmed by a Bartlett’s test of sphericity yielding a significance level of <0.0001. Table 1 delineates the NAQ outcomes in relation to sample characteristics, further segmented by geographic origin. Notably, the southern region displays notable variations in most independent variables. The age group of 20–30 years obtains the highest scores (117.42 ± 15.61) in contrast to the 61–70 years age group (100.08 ± 15.14) (*p* < 0.0001). Females exhibit a heightened receptivity to the nursing profession (111.18 ± 15.64) compared to their male counterparts (107.18 ± 15.14) (*p* = 0.022). Religious affiliation similarly impacts perceptions, with atheists displaying significantly higher scores (119.27 ± 15.23) than those identifying as Catholic (109.19 ± 16.00) (*p* < 0.0001). Among the occupation categories, students manifest a distinctly favorable perception (119.65 ± 14.32), followed by civil servants and private employees. Conversely, retirees present a markedly lower perception score of 98.30 ± 16.89 (*p* < 0.0001). Those within their family who have a relative practicing the profession exhibit a higher perception; the same applies to the southern sample that is aware of the nurse’s educational path. Regarding the north and the center, noteworthy discrepancies are absent, with the perception level largely echoing that observed in the south. However, deviations emerge in specific aspects. For instance, within the context of religion, the Catholic affiliation garners the highest scores. Additionally, in the north, retirees exhibit a heightened affinity for the nursing profession compared to other regions. Even satisfaction with the hospital care they received during their last admission appears to be significantly higher for citizens of northern Italy, with an average score of 116.24 ± 13.21 (*p* = 0.043). TV series, news reports, and experiences with nursing care during the COVID-19 pandemic seem to have had a limited impact on altering the perception of the nursing profession across various geographic regions. The observed effects on the image of nurses were not found to be statistically significant in any specific area.

The results of the NAQ, categorized by domain, exhibit significant disparities among the three geographic regions. When examining the overall NAQ scores, the north presents the highest values (114.64 ± 14.25), followed by the center (113.11 ± 17.11), and finally the south (113.11 ± 17.11) (*p* = 0.016). In the specific domain of the nurse’s professional role, no statistically significant differences are observed, with an overall mean score of 38.02 (SD = 6.08). However, variations emerge in the other domains of the NAQ:

1. Professional values of nurses are more prominently perceived in the north and central Italy (M = 26.27), and to a lesser extent in the south (25.06 ± 4.151) (*p* = 0.008);
2. Stereotypes are more prevalent in the north (23.73 ± 3.538) and the center (23.13 ± 4.104) (*p* = 0.001);
3. Professional activities are predominantly recognized in the north (16.69 ± 2.745) (*p* < 0.0001).

Interestingly, the only area where the south scores higher is in the domain of nurses’ characteristics (9.83 ± 2.570), followed by the north (9.52 ± 2.286) (*p* = 0.044) (as shown in Table 2).

The results reveal statistically significant differences across the three explored geographic areas. In question 2, beliefs about nurses’ protective role within the healthcare system vary by region. In the south, 78.5% (n = 317) hold this belief, followed by 77.8% (n = 35) in the center (*p* = 0.025). Concerning the importance of nursing services compared to physicians, 90.4% (n = 66) in the north and 84.4% (n = 38) in the center agree, while in the south, the agreement stands at 57.4% (n = 231) (*p* = 0.005). Disagreement regarding the potential reduction of nurses’ education is observed in the center (60.0%, n = 27) and the north (54.8%, n = 40), whereas 34.6% (n = 139) in the south disagree (*p* = 0.001). Perceptions about nurses’ potential for political engagement align in the center (48.9%, n = 22) and the south (43.0%, n = 168) (*p* < 0.001). Regarding nurses’ wages, dissatisfaction is higher in the north (63.0%, n = 46) and the center (62.2%, n = 28), compared to the south (35.6%, n = 138) (*p* < 0.001). Disagreement about the subordinate status of nurses to medical professionals is pronounced in the north (63.0%, n = 46) and the center (62.2%, n = 28), while only 35.6% (n = 138) in the south disagree (*p* < 0.001). The belief that the nursing profession is not merely a fallback to medicine is shared by 60.8% (n = 45) in the north and 59.1% (n = 26) in the center (*p* = 0.028). The importance of a university pathway for obtaining a nursing degree is agreed upon by individuals in the north (87.8%, n = 65), the center (73.3%, n = 33), and the south (72.2%, n = 285) (*p* = 0.042). Significant disagreement emerges regarding the notion that “One of the advantages of being a nurse is to marry a doctor”. Respondents in the north (95.9%, n = 71) express the highest level of disagreement, followed by the center (82.2%, n = 37), and the south (66.8%, n = 264) (*p* < 0.001) (as shown in Table 3). Regarding the summary questions “I would recommend a relative/acquaintance to be a nurse” and “I have an interest in choosing a nursing degree program,” agreement levels range from 33.3% to 57.1% among the sample.

Items with mean values above the 90th percentile corresponded to the following statements:

5. Nurses are a resource for people with health problems;

8. The service performed by nurses is as important as that provided by physicians.

Conversely, items with mean values below the 10th percentile corresponded to the following statements:

15. Nurses are sufficiently rewarded for their work by knowing that they are helping other people;

17. Nurses carry out the physician’s requests without objection;

19. Many nurses who seek advancement in their professional practice would rather be physicians;

23. Nurses are adequately paid for the work they perform;

27. One of the advantages of being a nurse is marrying a physician.

Respondents from northern Italy agreed with the highest values on items with values above the 90th percentile. The same pattern was observed with responses below the 10th percentile, except for the statement “Many nurses who seek advancement in their professional practice would rather be physicians”. The sample from central Italy indicated a value above the 90th percentile for the statement “Nurses are a resource for people with health problems”. However, this value was lower compared to the northern Italy sample, with a Δ = −0.16. Regarding items with values below the 10th percentile, respondents from central Italy expressed a value with a Δ = −0.19, indicating a higher level of disagreement. In the case of southern Italy, based on the parameters outlined in Table 3, the statement with a value above the 90th percentile is “The service performed by nurses is as important as that provided by physicians,” with a Δ = −0.23 compared to northern Italy. As for responses with values below the 10th percentile, the only item pertains to the response asking whether the only advantage of being a nurse is marrying a physician. In this case, the Δ = −0.74, indicating a higher level of disagreement. Table 4 presents the items and categories for which the greatest differences (below the 10th and above the 90th percentile) were observed among the three subgroups.

In terms of respondents’ general understanding of the nursing profession, no significant differences were observed across different regions of the country. A substantial 92.2% (n = 497) of the sample recognized the vital role nurses play within the entire Integrated Health System (IHS).

While 90.4% (n = 479) of respondents acknowledged the collaborative relationship between nurses and medical professionals, a noteworthy 47.2% (n = 249) still perceived nurses as secondary to the medical figure. However, a resounding 90.6% (n = 483) recognized the indispensability of nurses within the healthcare system, understanding that their role cannot be replaced by specialized social and health workers (94.5%, n = 499).

### 3.3. Associations

Pearson’s correlation coefficient revealed a consistent and positive correlation between the various domains of the NAQ and questions one, two, three, four, and six. This indicates that as the perception of the nurse’s image improved across these domains, there was a corresponding positive response to questions one, two, three, four, and six. On the contrary, item five, which conveys the notion of nurses as subordinate to doctors and the possibility of replacement by support staff, exhibited a negative correlation with all NAQ domains. Similarly, the question regarding the potential replacement of nurses with social and health workers also showed a negative correlation with all NAQ domains (as illustrated in Table 5).

## 4. Discussion

The study, aimed at assessing the image of nurses in Italy after the COVID-19 health emergency, revealed results largely consistent with the previous literature. The study’s outcomes align with a previous investigation, where the degree of perception among students educated through university orientation in nursing curriculum and functions was measured [13]. Interestingly, the current sample across all geographic regions showed overlapping scores with the aforementioned student group. This suggests that media portrayal during the COVID-19 pandemic, casting nurses as “heroes” within the healthcare system, might have influenced the respondents [14]. Both undergraduate and postgraduate nursing education have contributed to enhancing the visibility of the nursing profession, especially during the pandemic. This positive image is believed to have also positively impacted the quality of education for nursing professionals [15]. Over the years, nursing in Italy has transitioned from an auxiliary status to an intellectual profession, aided by legal decrees and access to various degree programs. Notably, the northern Italian sample emphasized the importance of bachelor’s and master’s degrees in nursing for enhancing patient care and treatment, aligning with the existing literature [16,17]. Across the entire population, ranging from north to south, there is recognition of the added value of undergraduate nursing education for nursing practice and scientific research [18,19]. The population acknowledges the significance of nursing research in improving patient care [20] and perceives nurses as indispensable within the healthcare system [21]. The characteristics and attitudes of humanization and personalized care associated with nursing correlate with gender stereotypes. These stereotypes contribute to the alignment of nursing with qualities often associated with the female gender, further driving individuals toward the nursing profession [14]. However, only half of the sample believes that men can excel as nurses, indicating potential direct or indirect influences from family members, past hospital experiences, or mass media during the pandemic [14]. The study underscores a reevaluation of stereotypes in favor of the nursing profession. The northern and central Italian samples indicated a critical stance against the concept of nurses carrying out physicians’ orders without objection. Interestingly, while the nursing profession is seen as autonomous by 66%, 81% perceive it as a job for intelligent individuals, and 83% consider it on par with the medical profession. These post-pandemic findings differ from previous studies [22,23,24], suggesting evolving perceptions. However, concerning overall professional respect, the population remained critical, returning to pre-pandemic values [9,25]. Despite the positive evidence, both the study sample and the literature highlight nursing as a profession with inadequate compensation [9,26,27,28,29]. Instances of disrespect, autonomy violations, underestimation of professional abilities, and denial of nurses’ value in care can lead to dignity violations, negatively impacting job satisfaction and performance [30,31]. Interestingly, only 38.2% of the sample, varying from 45.2% in the north to 20.5% in the center, perceived nurses as satisfied with their work. This finding is intriguing considering the increased demand for nursing services and stress experienced during the pandemic, juxtaposed with heightened nurse satisfaction from helping patients [32,33,34]. Stereotypes portraying nurses as subservient to physicians or replaceable by technical workers negatively correlate with NAQ domains. This suggests that the sample acknowledges the distinct competence of nurses, which is irreplaceable by other practitioners. The study population views nurses as integral to the healthcare system, collaborating with physicians, and recognizing that healthcare would be incomplete without nurses. These findings mirror what was highlighted in the literature during the initial pandemic phase [35]. Despite encouraging results regarding the image of nurses, responses diverge when asked about recommending nursing as a profession to relatives or friends. The sample’s responses are divided, with notable positivity observed in the south of Italy. The average Likert scores in these questions are similar to those of a comparable study among students exposed to nursing profiles and skills during a college orientation before the COVID-19 pandemic [13].

### Study Limitations

The present study is not without limitations. Foremost among these is the sampling method, which may not ensure a statistically significant representation of the entire Italian population. Additionally, the reliance on voluntary participation could introduce selection bias, as those who chose to participate may have different perspectives and experiences compared to those who opted not to participate. Another limitation could stem from the timeframe during which the data was collected, post-COVID-19, and from the use of the questionnaire in the Italian language, albeit previously employed in other studies [9,13].

## 5. Conclusions

The persistent stereotype of nurses as predominantly female, inclined towards nursing over a university career, and lacking full autonomy and authority in critical decision-making processes remains evident. This perception reflects the ongoing challenges and misconceptions that the nursing profession continues to face. Overall, the NAQ scores did not differ significantly across the three investigated geographic areas; however, some differences emerged in the domains and percentile values. The role and professional values were significantly emphasized in the northern and central Italy samples, where attributing nursing value equivalent to that of physicians and recognizing nurses as a resource for people with health problems were strongly endorsed. In terms of the domain exploring nurses’ characteristics, respondents from the south exhibited a greater sensitivity, as well as in the question where nurses and physicians were attributed the same importance. Citizens from northern and central Italy displayed greater sensitivity compared to the southern sample in believing that nurses are inadequately paid, do not aspire to become physicians, and actually view nursing as an autonomous practice. Their disagreement was notable in their perception of nurses as mere executors of physicians’ orders. Respondents from the south significantly agreed with those from the north that one advantage of being a nurse is not having to marry a doctor. Therefore, the conducted study highlighted an improved perception of the nurse’s image, likely due to the influence of mass media, familial connections with nurses, and previous hospitalizations, especially during the pandemic period. Despite these findings, the attractiveness of the nursing profession remains quite low. This could be attributed to the questionable level of respect that institutions have for the nursing field. The perception of inadequate salary, insufficient political representation, the prevailing association of nursing with the female gender being more inclined toward caregiving than a university career, the lack of respect, and an unsatisfactory nursing work environment all appear to influence decisions regarding recommending or pursuing a nursing education. Given the study’s findings, there is an urgent need to introduce educational initiatives, beginning at the school level, that emphasize the significance of nurses’ contributions within the national health service. The research demonstrates an improved perception of the nursing profession’s image, possibly attributed to media exposure, familial connections to nursing, and prior hospital experiences, particularly during the pandemic. Despite these positive shifts, the profession still struggles with a lack of attractiveness. This lack of allure appears to be connected to concerns regarding the profession’s standing within institutions. Perceptions of inadequate salaries, limited political representation, gender biases favoring nursing over higher education, insufficient respect, and subpar working conditions all play a role in influencing individuals’ decisions to recommend or pursue a nursing education. Addressing these underlying issues is crucial to enhancing the appeal of the nursing profession and ensuring its continued growth. Educational campaigns and structural changes within healthcare institutions are needed to promote nursing as a valued and respected profession, attracting a diverse range of individuals who can contribute to the betterment of healthcare services.

## Figures and Tables

**Table 1:** NAQ results according to sample characteristics.

	North		Center		South	
	n = 77		n = 45		n = 434	
	M ± DS	*p*	M ± DS	*p*	M ± DS	*p*
Age		0.23		0.072		<0.0001 **
20–30	115.79 ± 13.80		117.82 ± 13.18		117.42 ± 15.61	
31–40	113.65 ± 10.81		111.46 ± 19.75		109.15 ± 16.68	
41–50	115.55 ± 11.44		109.38 ± 12.54		105.68 ± 14.82	
51–60	105.80 ± 22.10		87.00 ± 38.18		107.09 ± 13.54	
61–70	124.50 ± 11.00				100.08 ± 15.14	
Gender		0.846		0.653		0.022 *
Male	115.24 ± 13.05		110.78 ± 22.89		107.18 ± 17.28	
Female	114.47 ± 14.67		113.69 ± 15.71		111.18 ± 15.64	
Religion		0.647		0.861		<0.0001 **
More	115.80 ± 9.96				103.95 ± 14.50	
Atheist	111.00 ± 19.26		115.00 ± 14.85		119.27 ± 15.23	
Christianity	115.77 ± 12.54		115.82 ± 12.20		109.19 ± 16.00	
Profession		0.67		0.006 **		<0.0001 **
Missing					97.57 ± 23.35	
Housewife			78.50 ± 28.99		107.20 ± 14.50	
Private employee	114.96 ± 11.51		109.90 ± 12.61		109.78 ± 14.70	
Civil servant	113.52 ± 17.23		112.47 ± 19.71		109.04 ± 15.85	
Unemployed	124.50 ± 2.12				107.83 ± 12.00	
Freelancer	109.56 ± 18.11		102.50 ± 0.70		103.50 ± 14.50	
Retired	128.00 ± 8.48				98.30 ± 16.89	
Student	115.50 ± 12.55		121.38 ± 9.48		119.65 ± 14.32	
Sample with nurse relatives		0.452		0.494		<0.0001 **
Yes	115.24 ± 12.78		114.06 ± 18.53		112.67 ± 16.22	
No	112.13 ± 19.55		109.80 ± 10.90		104.49 ± 14.61	
Degree of satisfaction last hospitalization		0.081		0.413		0.043
Satisfied	116.24 ± 13.21		107.44 ± 22.58		109.57 ± 15.74	
Dissatisfied	106.00 ± 20.90		110.00 ± 10.10		109.12 ± 14.13	
Neutral	/		117.00 ± 11.56		116.22 ± 20.34	
Respondents who received nursing care during the COVID-19 pandemic		0.222		0.123		0.939
Yes	115.16 ± 12.92		107.53 ± 23.61		110.26 ± 15.02	
No	114.38 ± 14.96		115.90 ± 12.29		110.40 ± 16.70	
To what extent did TV series and/or news reports influence her image of the nurse		0.385		0.892		0.085
Nothing	114.38 ± 13.44		114.67 ± 19.81		110.64 ± 16.12	
Somewhat	117.68 ± 11.07		110.21 ± 20.77		112.97 ± 14.94	
Quite	111.46 ± 18.72		114.47 ± 10.64		110.91 ± 15.74	
Very	/		/		104.98 ± 16.98	
Knows the educational background of the nurse practitioner	0.546		0.113		<0.0001 **	
Yes	115.06 ± 14.52		115.14 ± 18.19		115.00 ± 16.19	
No	112.33 ± 13.05		105.00 ± 8.39		103.46 ± 13.27	

* *p* = < 0.05; ** *p* = < 0.01.

**Table 2:** NAQ results by domain (‡).

			Missing	North	Center	South	Total		
			n = 8	n = 77	n = 45	n = 434			
	Item	R			M ± DS			F	*p*
Professional role	10	10–50	34.50 ± 5.682	38.43 ± 5.033	39.20 ± 6.490	37.89 ± 6.197	38.02 ± 6.08	1.64	0.177
Professional values	7	7–35	22.25 ± 2.375	26.27 ± 4.312	26.27 ± 5.024	25.06 ± 4.151	25.28 ± 4.26	3.99	0.008 **
Stereotypes	6	6–30	19.63 ± 3.292	23.73 ± 3.538	23.13 ± 4.104	21.97 ± 4.165	22.27 ± 4.12	5.87	0.001 **
Professionalism	4	4–20	13.50 ± 2.976	16.69 ± 2.745	15.78 ± 3.176	15.23 ± 2.906	15.45 ± 2.95	6.86	<0.0001 **
Characteristics of nurses/nursing	3	3–15	9.63 ± 2.200	9.52 ± 2.286	8.73 ± 2.580	9.83 ± 2.570	9.70 ± 2.54	2.71	0.044 *
I would advise a relative/acquaintance to be a nurse	1	1–5	3.43 ± 0.787	3.22 ± 1.261	3.27 ± 1.370	3.54 ± 1.115	3.47 ± 1.160	2.080	0.102
I have an interest in choosing a nursing degree program	1	1–5	2.33 ± 1.528	2.79 ± 1.554	3.18 ± 1.690	3.12 ± 1.558	3.06 ± 1.571	1.129	0.337

* *p* = < 0.05; ** *p* = < 0.01. ‡ = Domains composing the NAQ recommended by Toth et al. [12].

**Table 3:** Percentage of agreement by individual NAQ question.

Missing			North		Center		South		Total			
	n	%	n	%	n	%	n	%	N	%	X^2^	*p*
1. Nurses are those who advocate for patients’ rights	4	57.1%	42	56.8%	29	67.4%	288	71.1%	363	68.6%	9.85	0.131
2. Nurses protect patients within the health care system	4	57.1%	52	69.3%	35	77.8%	317	78.5%	408	76.8%	2.80	0.025 *
3. Nurses participate in the development of policies on health care	4	57.1%	44	60.3%	32	71.1%	294	73.9%	374	71.5%	7.23	0.300
4. Nurses must wear a white uniform to be identified	4	57.1%	28	37.8%	13	28.9%	180	44.9%	225	42.7%	7.50	0.277
5. Nurses are a resource for people with health problems	6	85.7%	69	92.0%	40	88.9%	341	84.2%	456	85.7%	6.34	0.387
6. Nurses are generally kind and compassionate	6	85.7%	40	54.1%	20	45.5%	252	61.9%	318	59.8%	9.05	0.171
7. Being a nurse requires intelligence	5	71.4%	66	89.2%	39	88.6%	321	79.3%	431	81.3%	12.2	0.057
8. The service performed by nurses is as important as that provided by physicians	5	71.4%	66	90.4%	38	84.4%	333	82.2%	442	83.4%	18.6	0.005 **
9. If nurses spent more time caring for patients and less time at the university, everyone would benefit	3	42.9%	16	21.9%	10	22.2%	153	38.1%	182	34.5%	22.1	0.001 **
10. Nurses integrate health teachings into nursing practice	4	57.1%	59	80.8%	33	75.0%	312	78.4%	408	78.2%	5.23	0.514
11. Scientific research is vital to the nursing profession	4	57.1%	62	82.7%	33	75.0%	303	75.2%	402	76.0%	7.58	0.270
12. Nurses are politically active	3	42.9%	28	38.4%	22	48.9%	168	43.0%	221	42.8%	30.0	<0.001 **
13. Nurses can operate independently	3	42.9%	50	66.7%	34	75.6%	255	64.7%	342	65.6%	7.59	0.270
14. Nurses speak out sharply against inadequate working conditions	3	42.9%	40	54.8%	15	34.1%	206	53.5%	264	51.9%	12.8	0.046 *
15. Nurses are sufficiently rewarded for their work by knowing that they are helping other people	3	42.9%	7	9.5%	6	13.6%	116	29.4%	132	25.4%	37.1	<0.001 **
16. Nurses must have the right to strike	4	57.1%	53	73.6%	34	75.6%	291	73.1%	382	73.2%	6.73	0.347
17. Nurses carry out the physician’s requests without objection	3	42.9%	12	16.4%	6	13.3%	112	28.9%	133	25.9%	28.9	<0.001 **
18. Men make good nurses	4	57.1%	48	65.8%	22	48.9%	210	53.3%	284	54.7%	4.99	0.545
19. Many nurses who seek advancement in their professional practice would rather be physicians	3	42.9%	13	17.6%	4	9.1%	84	21.5%	104	20.2%	14.1	0.028 *
20. Nursing work is exciting	4	57.1%	59	79.7%	30	68.2%	258	65.5%	351	67.6%	6.69	0.351
21. Nurses integrate scientific research findings into clinical practice	4	57.1%	52	70.3%	29	64.4%	252	64.8%	337	65.4%	3.14	0.791
22. The most important goal of nursing research is to improve patient care	4	57.1%	66	88.0%	33	73.3%	298	74.9%	401	76.4%	9.81	0.133
23. Nurses are adequately paid for the work they perform	3	42.9%	3	4.1%	5	11.1%	73	18.4%	84	16.1%	27.2	<0.001
24. Nurses value the time they spend at patients’ bedsides caring for them	4	57.1%	54	72.0%	25	55.6%	233	60.1%	316	61.4%	11.2	0.082
25. Nurses must have a bachelor’s degree to practice professionally	4	57.1%	65	87.8%	33	73.3%	285	72.2%	387	74.3%	13.0	0.042 *
26. Nurses with master’s degrees make important contributions to patient care	4	57.1%	52	71.2%	22	48.9%	218	55.8%	296	57.4%	16.5	0.011 *
27. One of the advantages of being a nurse is marrying a physician	3	42.9%	2	2.7%	1	2.2%	43	10.9%	49	9.4%	40.2	<0.001 **
28. Nursing is a respected profession	3	42.9%	24	32.4%	8	17.8%	124	31.3%	159	30.5%	10.9	0.091
29. Nurses constantly update their practice in relation to the latest findings on health care	3	42.9%	41	55.4%	25	56.8%	217	55.2%	286	55.2%	10.4	0.110
30. Nurses are satisfied with the work they do	3	42.9%	33	45.8%	9	20.5%	152	38.7%	197	38.2%	19.8	0.003 **
Summary question 1. Would I advise a relative/acquaintance to be a nurse	4	57.1%	33	45.2%	19	43.2%	212	53.9%	268	51.8%	15.6	0.016 *
Summary question 2. I have an interest in choosing a nursing degree program	1	33.3%	26	35.6%	19	50.0%	124	44.0%	170	42.9%	4.19	0.652

* *p* = <0.05; ** *p* = <0.01.

**Table 4:** Highest- and lowest-attitude scores.

Item	Lowest-Value Category	Highest-Value Category
Upper 10%		
8. The service performed by nurses is as important as that provided by physicians	South (4.30 ± 0.93)	North (4.53 ± 0.80)
5. Nurses are a resource for people with health problems	Center (4.40 ± 0.96)	North (4.56 ± 0.74)
Lower 10%		
27. One of the advantages of being a nurse is marrying a physician	North (1.22 ± 0.66)	South (1.96 ± 1.24)
23. Nurses are adequately paid for the work they do	North (1.84 ± 0.98)	Center (1.91 ± 1.16)
15. Nurses are sufficiently rewarded for their work by knowing that they are helping other people	North (1.96 ± 1.13)	Center (2.20 ± 1.17)
17. Nurses carry out the physician’s requests without objection	North (2.22 ± 1.18)	Center (2.24 ± 1.19)
19. Many nurses who seek advancement in their professional practice would rather be physicians	Center (2.11 ± 1.17)	North (2.28 ± 1.14)

**Table 5:** Correlation of NAQ areas with items on general knowledge of the nurse.

	Professional Role	Professional Values	Stereotypes	Professionalism	Characteristics of Nurses
The nurse plays a crucial role in the entire national health service	0.415 **	0.239 **	0.255 **	0.283 **	0.163 **
The nurse is the professional who works in full cooperation with the physician	0.342 **	0.123 **	0.146 **	0.232 **	0.177 **
Health care would not function without nurses	0.345 **	0.208 **	0.197 **	0.256 **	0.150 **
The nurse is a professional who works as a function of the physician	−0.139 **	−0.350 **	−0.337 **	−0.242 **	0.185 **
The nurse can be replaced by the figure in the Health Care Worker Spec	−0.194 **	−0.131 **	−0.118 **	−0.080	0.090 *
I would advise a relative/acquaintance to be a nurse	0.312 **	0.240 **	0.292 **	0.325 **	0.595 **
I have an interest in choosing a nursing degree program	0.436 **	0.448 **	0.443 **	0.268 **	0.285 **

* *p* = <0.05; ** *p* = <0.01.

## Data Availability

Data are available from the first author upon reasonable request.

## Public Involvement Statement

The present study was approved by the bioethics committee of the University of Bologna with prot. 13221 of 8 February 2017. Adherence was spontaneous through a special acceptance command on the platform questionnaire.

## Guidelines and Standards Statement

STROBE checklist.
